# Author Correction: Dual phase patterning during a congruent grain boundary phase transition in elemental copper

**DOI:** 10.1038/s41467-023-36850-0

**Published:** 2023-03-02

**Authors:** Lena Langenohl, Tobias Brink, Rodrigo Freitas, Timofey Frolov, Gerhard Dehm, Christian H. Liebscher

**Affiliations:** 1 Max-Planck-Institut für Eisenforschung GmbH, Max-Planck-Straße 1, 40237 Düsseldorf, Germany; 2 Department of Materials Science and Engineering, Massachusetts Institute of Technology, Cambridge, MA 02139 USA; 3 Lawrence Livermore National Laboratory, Livermore, CA 94550 USA

**Keywords:** Phase transitions and critical phenomena, Surfaces, interfaces and thin films

Correction to: *Nature Communications* 10.1038/s41467-022-30922-3, published online 09 June 2022

Since the publication of this work, Lena Langenohl has changed her name from Lena Frommeyer. This has now been amended in the HTML and PDF versions of the article.

