# Supplementary material for: Dual phase patterning during a congruent grain boundary phase transition in elemental copper
Source: Nat Commun. 2022 Jun 9;13:3331. doi: 10.1038/s41467-022-30922-3 (PMC9184537; doi:10.1038/s41467-022-30922-3)
Supplement: Supplementary file 1 — Supplementary Information [file 41467_2022_30922_MOESM1_ESM.pdf]

Supplementary Information

Dual phase patterning during a congruent grain boundary phase transition in elemental copper

Lena Frommeyer, Tobias Brink,
Rodrigo Freitas, Timofey Frolov,
Gerhard Dehm, and Christian H. Liebscher

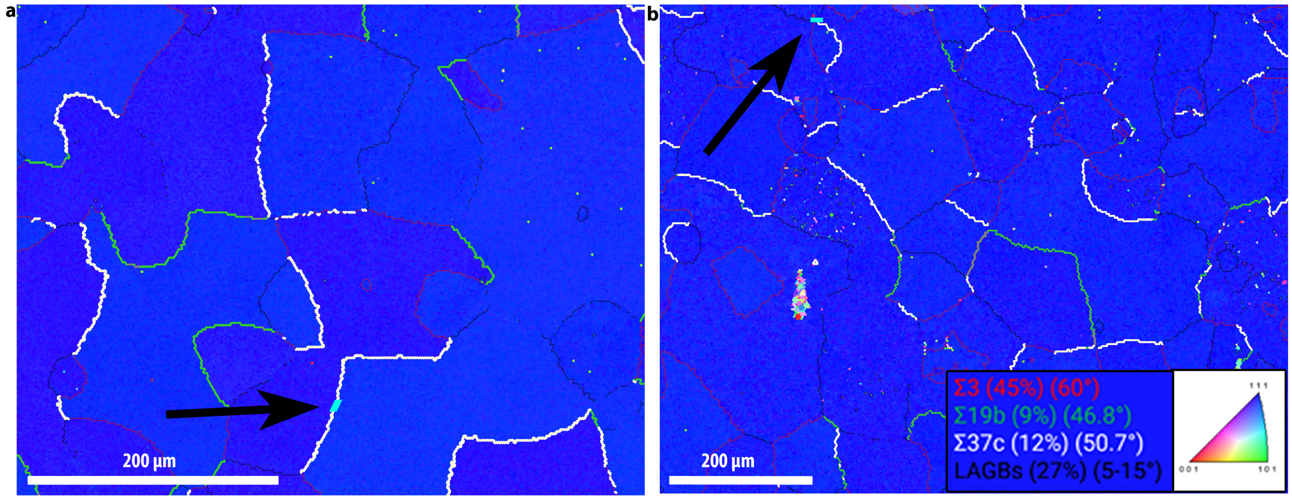

Supplementary Figure 1: Inverse pole figure maps from electron backscatter diffraction scans at different regions of the investigated Cu thin film. All grains have a ⟨111⟩ orientation normal to the image plane with a deviation of max. 2°. The grain size is between 100 µm and 300 µm. The perfect misorientation angles between the grains are indicated in the table as well as the total fraction of each grain boundary occuring in the Cu film. The transmission electron microscopy images shown in this paper are images of lamellas which have been lifted out at positions highlighted by cyan rectangles in (a) and (b) which are indicated with big arrows.

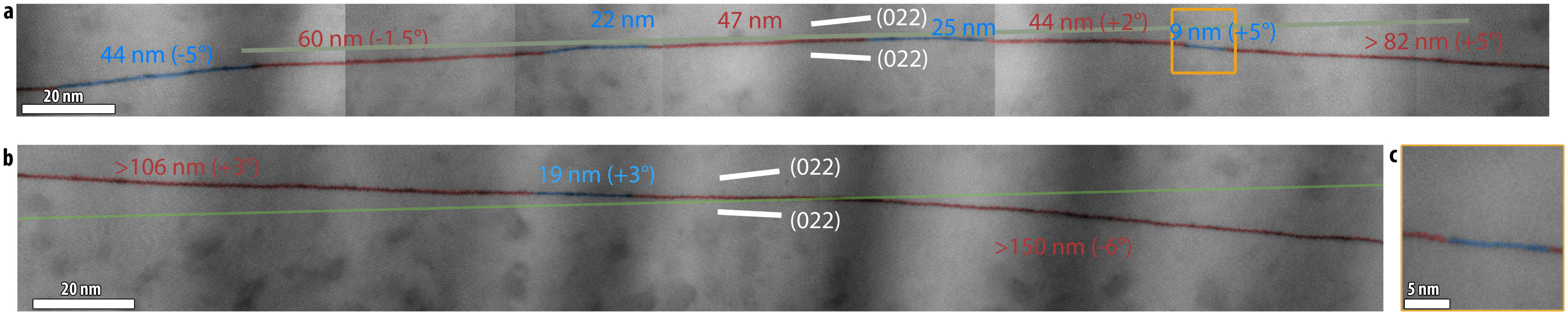

Supplementary Figure 2: Full resolution version of Fig. 2 from the main text: Overviews of two, more than 300 nm long, GB segments assembled from multiple HAADF-STEM images of near-symmetric areas of a Σ37c ⟨111⟩ GB. (a)–(b) The GBs consist of multiple, alternating domino (red) and pearl (blue) segments. The lengths of each segment are indicated as well as the deviation off the symmetric GB plane (green line). (c) Magnified view of the orange region in (a). The domino segments in symmetric areas (less than 5° off the symmetric case) are between 40 and 60 nm long, whereas the pearl segments are shorter with 10 nm to 40 nm.

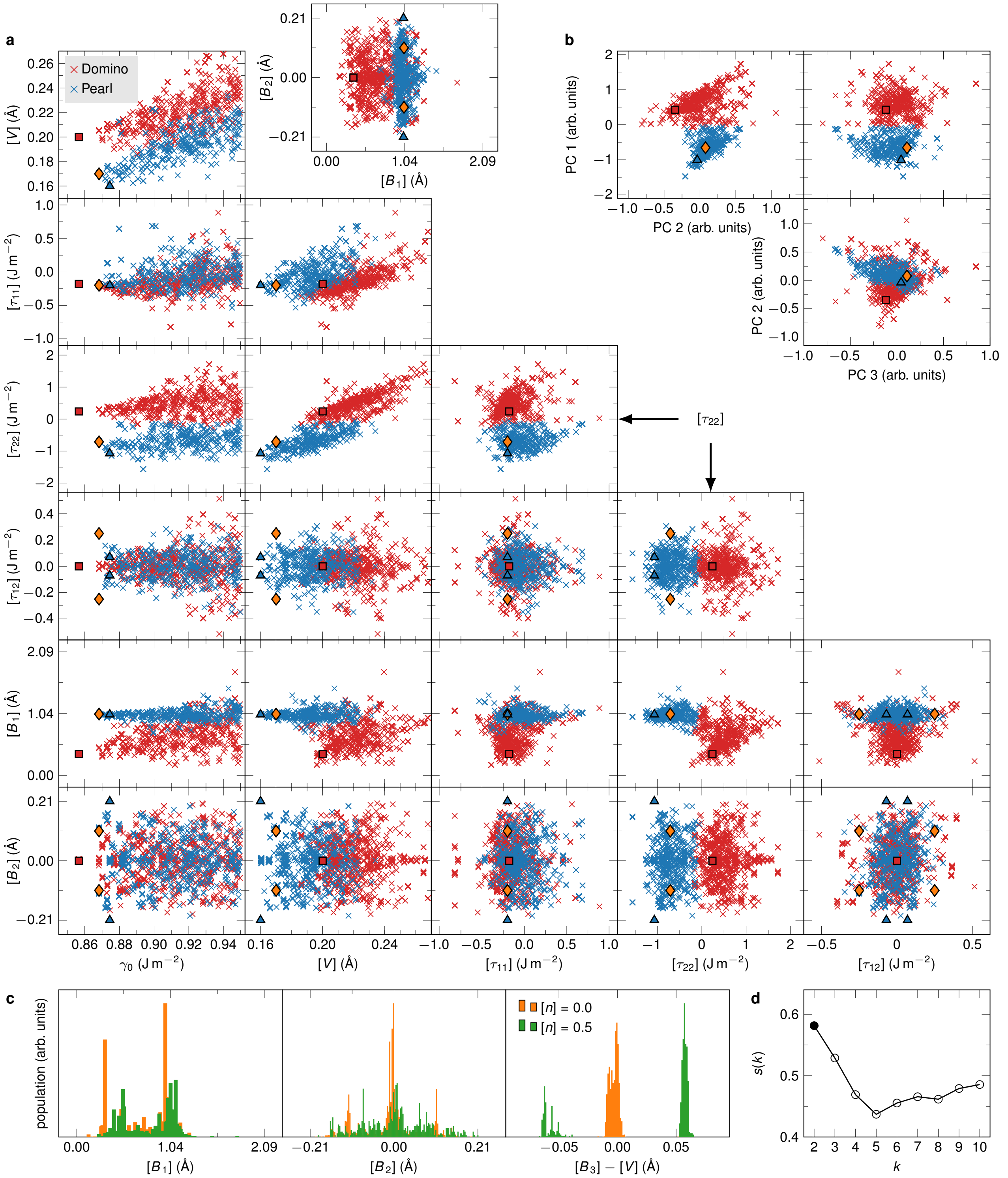

Supplementary Figure 3: (a) All possible pair plots of GB excess properties, clustered into $k = 2$ clusters. Markers for the perfect structures correspond to Fig. 4(a) in the main text. (b) Principal component (PC) analysis also highlights the clear separation of the data into two clusters. (c) In this GB, the excess number of atoms $[n]$ does not play a role, here indicated by histograms of the components of $[\mathbf{B}]$, separated into the different observed values of $[n]$. The same values occur, except for the component normal to the GB, $[B_3]$. Here, the interstitial-type or vacancy-type defect gives an additional shift not reflected in the excess volume. (d) The silhouette coefficient for different numbers of clusters k. A silhouette coefficient close to 1 means that the clusters are well separated and we see here that $k = 2$ is the best choice.

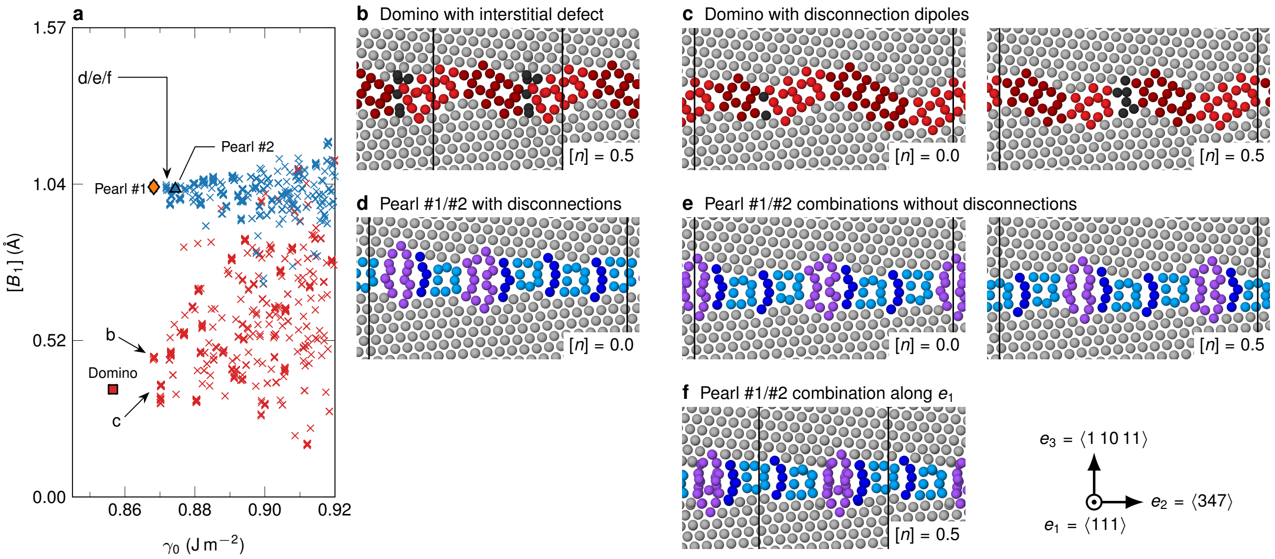

Supplementary Figure 4: Low-energy defective variants of the structures. (a) Several low-energy structures that have not been shown in Fig. 4 in the main text are indicated on this plot of the microscopic translation in tilt direction $B_1(\gamma_0)$. These are defective structures: (b) This structure is simply a domino phase with an interstitial-type defect. (c) Otherwise, simulation cells with disconnection dipoles can be found for domino. (d) A mixture of the two pearl variants with an added disconnection dipole, visible by the disturbed pattern of the square motifs (|Ω Ƨ B S Ƨ S| instead of |Ω Ƨ S B Ƨ S|; P motifs omitted for clarity). (e–f) Pearl #1 and #2 motifs can also combine without clear disconnections, both with and without point defects ($[n]$), due to the very small Burgers vector between them. The combination can occur by alternating motifs along the e_2 direction (e) or by stacking along the e_1 direction (f, cf. Supplemental Fig. 7). Black lines indicate the simulation cell.

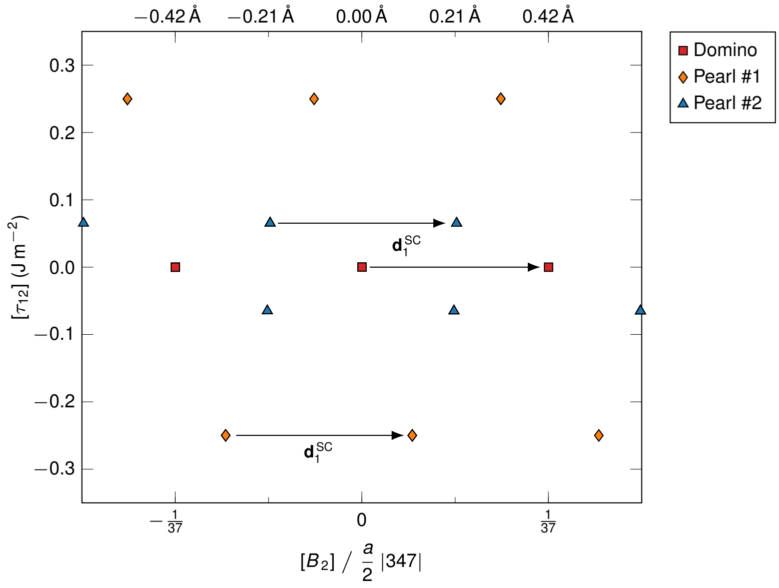

Supplementary Figure 5: Dependence of $[\tau_{12}]$ on $[B_2]$ for the different phases. For pearl #1 the sign of $[\tau_{12}]$ depends on $[B_2]$, while pearl #2 occurs in two otherwise equivalent variants only differing in the sign of $[\tau_{12}]$.

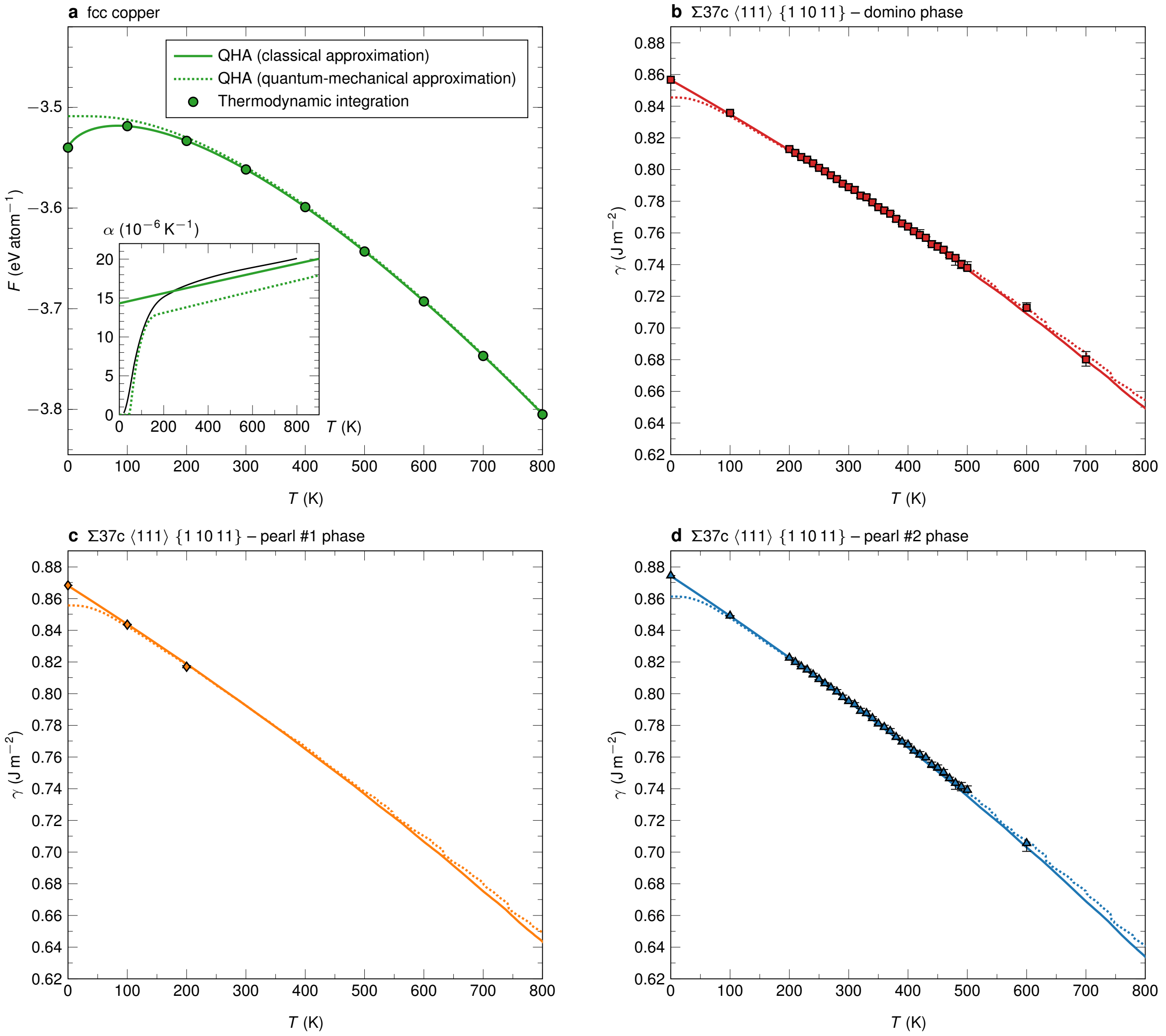

Supplementary Figure 6: Comparison of quasi-harmonic approximation (lines) and thermodynamic integration (data points) using an EAM potential. (a) Free energy of fcc copper. The inset shows the thermal expansion coefficient. The black line is experimental data from Hahn, J. Appl. Phys. **41**, 5096 (1970). (b)–(d) GB free energies for the three low-energy structures. (c) In pearl #1 thermodynamic integration simulations over 200 K, the pearl #2 B motifs start growing, invalidating the thermodynamic integration results, which are consequently not shown in the graph. This is expected, since pearl #2 has lower free energy than pearl #1 at higher temperatures and the transition likely has a very low energy barrier.

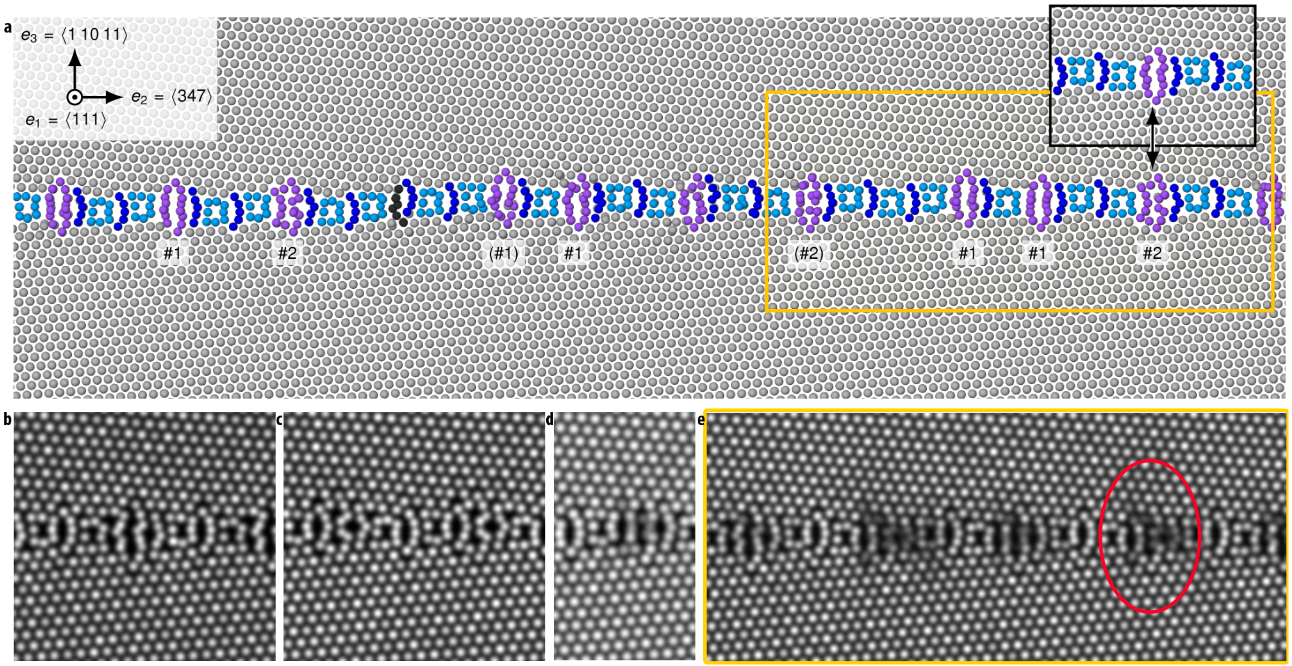

Supplementary Figure 7: (a) Simulation from Fig. 6b in the main text after continuing annealing to a total of 2 ns at 800 K so that all domino phase disappeared, cooling to 400 K with a cooling rate of 10^{11} K s^{-1}, and finally performing an energy minimization. Slice of width 1 nm, inset shows a slice 1 nm higher in ⟨111⟩ direction. STEM-Simulations of (b) pure pearl #1 structure, (c) pure pearl #2 structure, and (d) a mixture of pearl #1 on top of pearl #2 on structures obtained with the USPEX simulations. (e) STEM-Simulation of the indicated region of the simulation cell shown in (a), using the total height of 6.3 nm in ⟨111⟩ direction of the original simulation cell. The red circle marks the area where the stacked B and Ω motifs occur as indicated in the inset of (a).

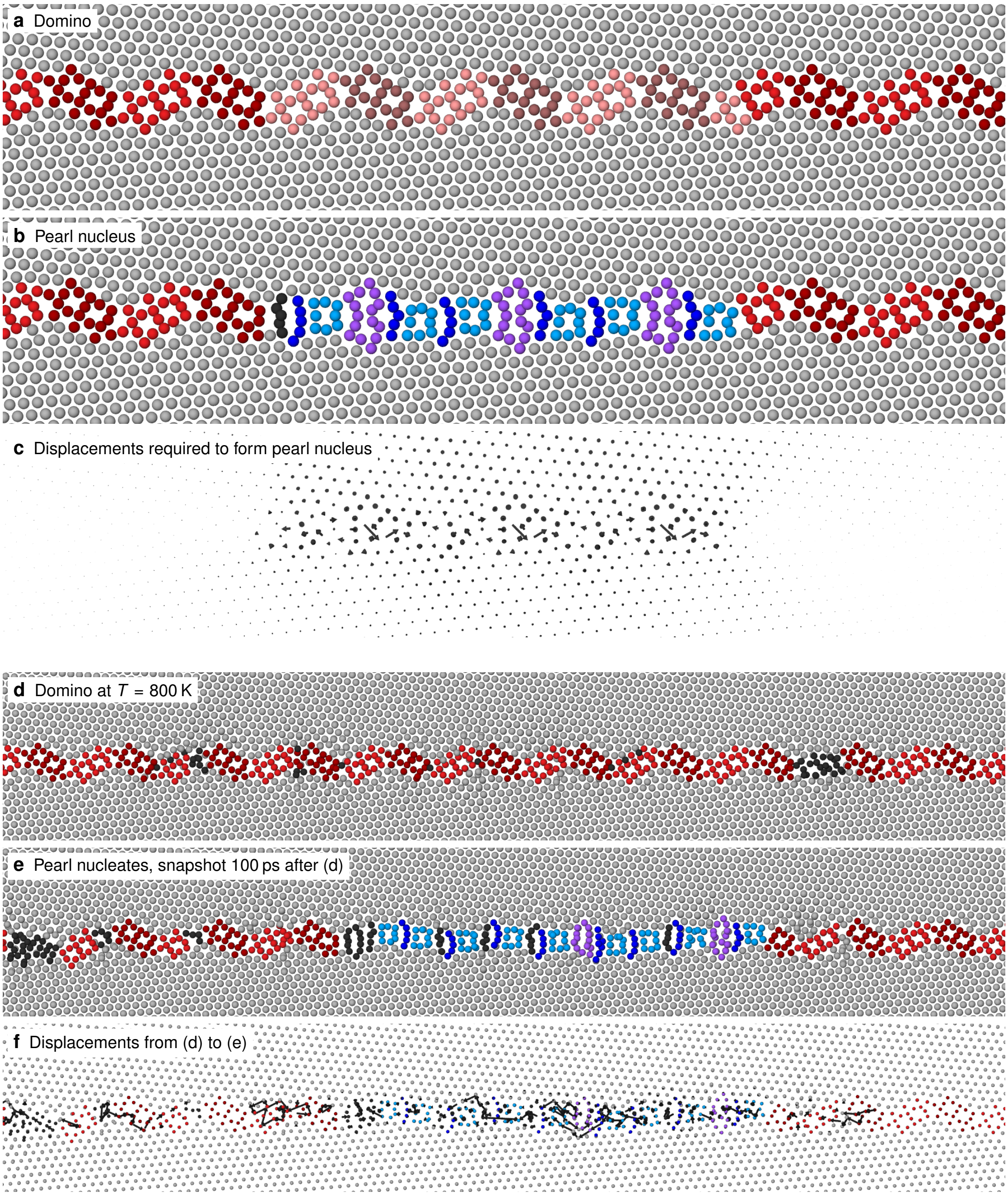

Supplementary Figure 8: Atomic shuffling required for the GB phase transition. (a)–(c) Minimal required displacements to transform domino into pearl. We produced two simulation cells, one with only the domino phase (a) and one where we inserted a pearl nucleus (b). We then calculated the smallest displacements between these cells, displayed as arrows in panel (c). The simulations show that displacements on the order of 1 Å are required, except for the B motifs, where two jumps of approximately 2 Å take place. No long-range diffusion is necessary for this phase transition. (d)–(f) We also analysed the simulation from Fig. 6(b) in the main text in the same way. There are more atomic jumps, but they still take place over comparatively short distances, also appear in the untransformed domino phase, and parts of the pearl phase exhibit similar jumps as in panel (c). The longer jumps thus represent either self diffusion due to the high temperature or are connected to disconnection nucleation and movement. Panels (d)–(f) show slices of width 1 nm.

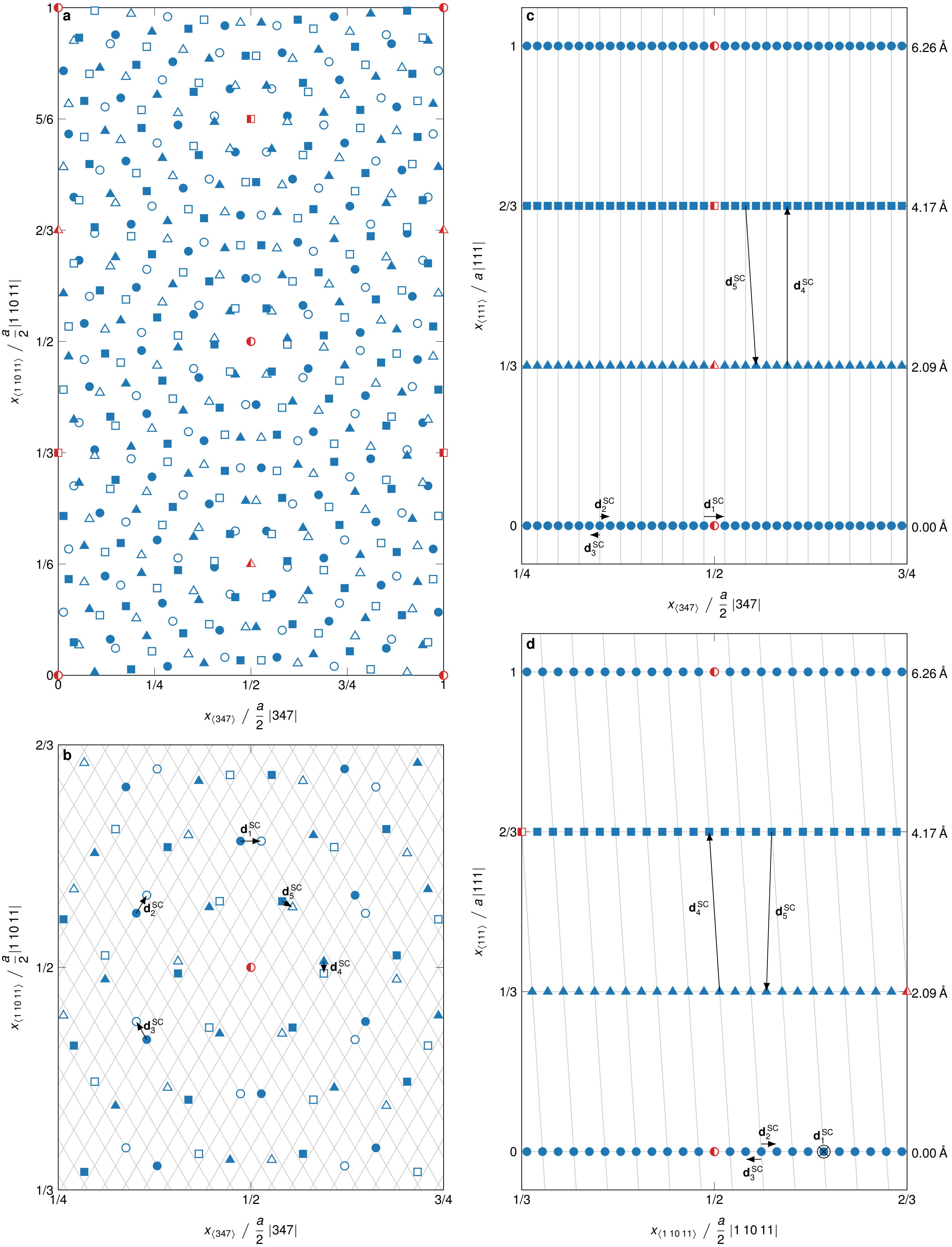

Supplementary Figure 9: Dichromatic pattern, with filled and unfilled symbols representing atomic positions of the two grains, respectivley. (a) Full CSL unit cell viewed from the tilt axis direction. (b)–(d) Zooms on the dichromatic pattern from different viewing directions. Some DSC vectors and the DSC lattice (grey lines) are shown. The lengths on the right are for the 0 K lattice constant of the EAM potential, $a = 3.615$ Å.

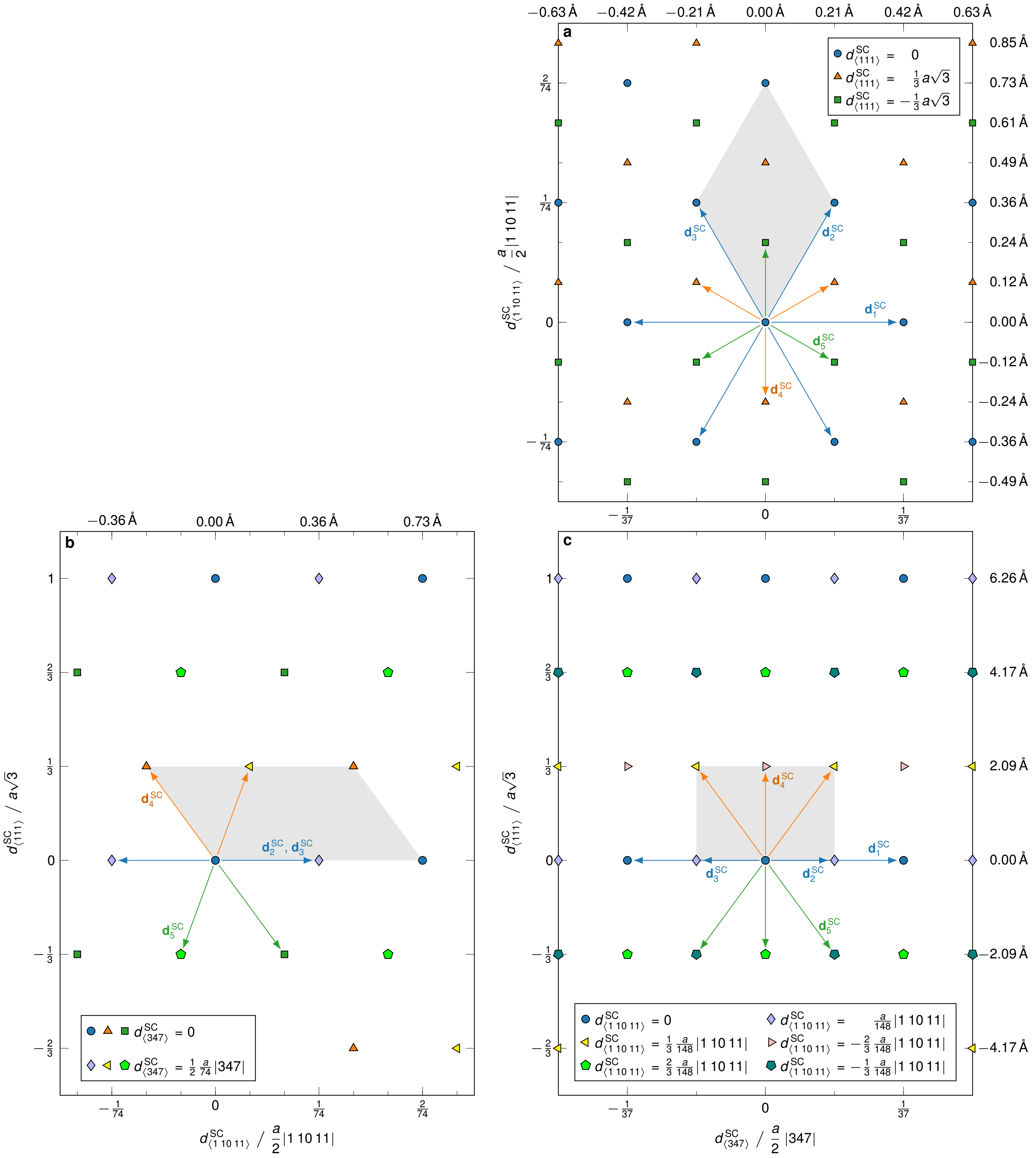

Supplementary Figure 10: Possible values of the DSC vectors. Gray areas represent the DSC unit cell, which is described by its basis vectors $\mathbf{d}_2^{\mathrm{SC}}$, $\mathbf{d}_3^{\mathrm{SC}}$, and $\mathbf{d}_4^{\mathrm{SC}}$. (a) View from the tilt direction. (b) View along the GB. (c) View along the GB normal. The lengths on top and right are for the 0 K lattice constant of the EAM potential, $a = 3.615$ Å.

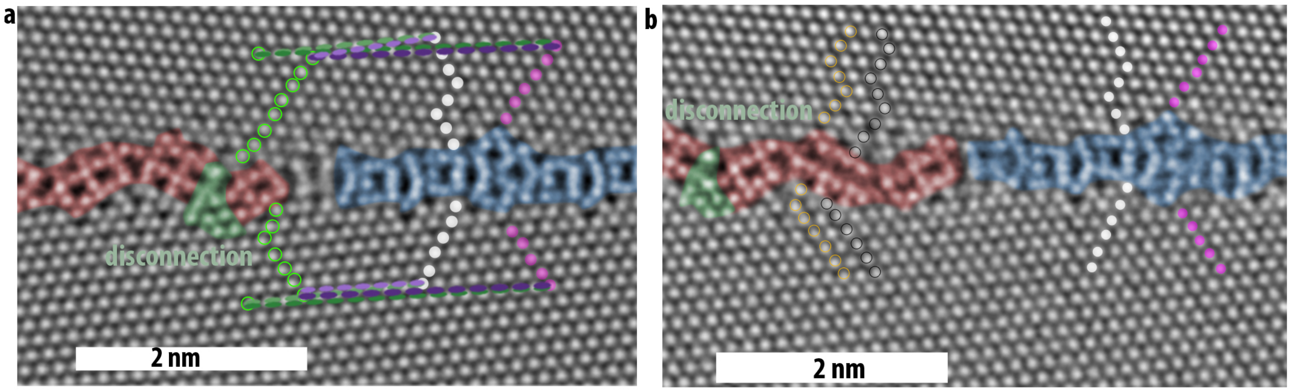

Supplementary Figure 11: Burgers circuits at two different phase junctions. The Burgers vector of the phase junctions in (a) is $(?, 0.33 \text{ to } 0.48, 0.05 \text{ to } 0.20)$ Å. The Burgers vector of the phase junctions in (b) is $(?, 0.25 \text{ to } 0.38, 0.05 \text{ to } 0.26)$ Å. The disconnections close to the phase junctions are $\mathbf{b}_{\text{exp., disc}} = (?, 0.23, -0.06)$ Å.
